# Opportunities and challenges of conversion type cathodes in zinc sulfur and zinc iodine batteries

**DOI:** 10.1016/j.isci.2025.114462

**Published:** 2025-12-27

**Authors:** Wenfang Li, Wenting Kong, Benjamin Tawiah, Hao Jia

**Affiliations:** 1 Key Laboratory of Eco-Textiles, Ministry of Education, Jiangnan University, Wuxi 214122, China; 2 Department of Industrial Art (Textiles), Kwame Nkrumah University of Science and Technology, PMB, Kumasi AK-417-4732, Ghana

**Keywords:** Energy engineering, Energy systems, Energy storage

## Abstract

Conventional cathode materials for zinc-ion batteries (ZIBs) predominantly rely on ion-insertion electrochemistry, which inherently limits their specific capacity and restricts the full realization of ZIBs’ performance potential. In contrast, cathode materials based on conversion reactions offer a promising pathway toward achieving higher energy densities. Among them, zinc-sulfur (Zn-S) and zinc-iodine (Zn-I_2_) batteries have attracted considerable attention for their commercial viability, yet targeted reviews addressing their reaction mechanisms, recent advances, and current challenges remain relatively scarce. To address this gap, this review provides a concise overview of the reaction mechanisms and battery configurations pertinent to conversion-type cathodes in Zn-S and Zn-I_2_ systems. Furthermore, it critically examines the fundamental physicochemical and structural challenges inherent to these materials, along with an evaluation of engineered solutions and emerging technological innovations designed to overcome these limitations. Finally, it outlines prospective research directions and development opportunities for advancing conversion-type cathodes in next-generation ZIBs.

## Introduction

Aqueous zinc-ion batteries (ZIBs) have gained considerable attention as a promising alternative to lithium-ion batteries (LIBs), motivating extensive research into high-performance cathode materials.^1^^,^^2^^,^^3^^,^^4^^,^^5^^,^^6^ Conventional intercalation-type cathodes are characterized by an open and stable crystal lattice, which contains interconnected channels or interlayer spacings that allow reversible, rapid, and stable insertion/extraction of zinc (Zn) ions without significant structural collapse, thereby delivering excellent cycling stability.^7^ In contrast, conversion-type cathodes store energy via a “conversion reaction” with Zn^2+^ ions, which generally enables higher specific capacity and energy density, demonstrating considerable application potential.^8^^,^^9^^,^^10^ Moreover, conversion-type cathode materials commonly used in ZIBs, such as sulfur (S), iodine (I_2_), and bromine (Br_2_), offer significant benefits over intercalation-type materials in terms of resource abundance and cost-effectiveness.^11^^,^^12^^,^^13^^,^^14^ Among these, Zn-S batteries and Zn-I_2_ batteries have garnered particular attention.^15^^,^^16^^,^^17^^,^^18^ Although both are applied in ZIBs, they exhibit significant differences in charge storage mechanisms, operating voltage, specific capacity, energy density, and cycle stability. Zn-S batteries offer a high theoretical specific capacity (≈1670 mAh/g) and theoretical energy density (≈400 Wh·kg^−1^).^19^^,^^20^ In contrast, Zn-I_2_ batteries are characterized by a relatively high and stable operating voltage (≈1.28 V vs. Zn^2+^/Zn) and considerable theoretical energy density (≈250 Wh/kg), making them well-suited for low-cost, low-power applications.^21^ The prospects for these two conversion-type cathodes hinge on overcoming their inherent reaction challenges, which would position them as highly competitive candidates for large-scale energy storage.^22^^,^^23^ In fact, conversion-type cathodes have been extensively studied in other battery systems, including LIBs and sodium-ion batteries, with several reviews elucidating their reaction mechanisms and research progress across these platforms.^24^^,^^25^^,^^26^^,^^27^^,^^28^^,^^29^ Although these features are promising and several notable studies on conversion reaction-based ZIBs have been published, there is still a lack of systematic overviews and in-depth discussions regarding these developments.

Given the growing research interest, it is essential to identify the shortcomings of Zn-S and Zn-I_2_ battery technologies to prevent misapplication in unsuitable scenarios. While challenges associated with the Zn metal anode (dendrite growth, hydrogen evolution, and corrosion) are acknowledged, they are beyond the present scope, focusing on conversion-type cathodes.^30^ Both S and I_2_ cathodes urgently demand robust strategies that suppress the dissolution and shuttling of redox-active species^31^; a breakthrough achieved in one platform is immediately translational to the other. A systematic comparison of their electrocatalytic-activity requirements and reaction-pathway regulation not only deepens the mechanistic understanding of conversion cathode chemistry but also establishes universal design principles for high-performance catalysts. Cross-disciplinary advancements in host architecture design, catalytic site engineering, separator functionalization, electrolyte formulation, and multi-electron reaction strategies are accelerating the development of Zn-S and Zn-I_2_ batteries toward commercial viability.

## Working principles and battery configurations

A key commonality between Zn-S and Zn-I_2_ batteries is that both systems are rooted in multi-electron transfer conversion reactions, which stem from their intrinsic physicochemical properties as shown in Table 1.^32^

**Table 1:** Physicochemical properties of S and I elements

Elements	Mole Mass (g mol^−1^)	Mass Density (g cm^−3^)	Melting Point (°C)	Boiling Point (°C)	Conductivity (S cm^−1^)	Capacity (mAh g^−1^)	Theoretical Voltage (V, Zn^2+^/Zn)
S	32.1	2.07	115.21 (α-S)	444.6	>×10^−30^	1675	0.26
I	126.9	4.99	113.7	184.3	>×10^−6^	211	1.30

**Figure 1:**
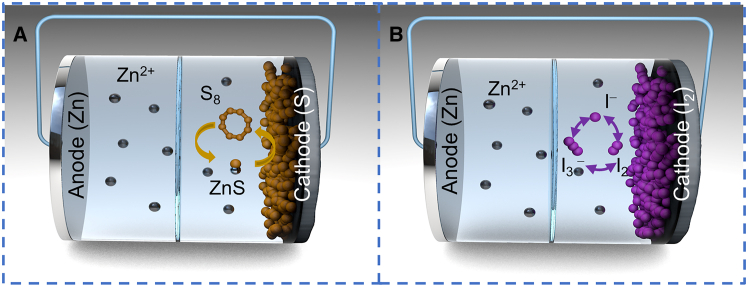
Schematic diagram of reaction mechanisms for Zn-S (A) and Zn-I_2_ (B) batteries

At the cathode, sulfur (S_8_/S^2−^) or iodine (I_2_/I^−^) undergo reversible redox reactions that involve dissolution, deposition and electron transfer, while Zn metal anode undergoes reversible plating and stripping as shown in Figure 1. However, they differ in specific details. The detailed working principles and reaction mechanisms of Zn-S and Zn-I_2_ batteries are as follows:

The Zn-S battery, composed of an elemental sulfur cathode, Zn metal anode, and mild electrolyte, primarily undergoes a two-electron solid-solid redox reaction during the discharge process.^33^ During discharging, the Zn metal anode loses electrons and is oxidized to Zn^2+^, whereas the elemental S cathode gains electrons and combines with Zn^2+^ to form ZnS. During charging, the reaction proceeds in the reverse direction, thus ZnS loses electrons at the cathode to be converted back into elemental S and Zn^2+^, and Zn^2+^ gains electrons at the anode to be reduced to Zn metal. The reaction mechanism can be briefly summarized as follows:

(1) $$\text{Anode}:\text{Zn}-2e^{-}↔Zn^{2+}$$

(2) $$\text{Cathode}:S+2e^{-}+Zn^{2+}↔\text{ZnS}$$

(3) Overall:Zn+S↔ZnS

Currently, Zn-I_2_ batteries are primarily composed of Zn metal anode, I_2_-containing cathode, and mild electrolyte. Their electrochemical reactions proceed based on the conversion of the I_2_/I^−^ redox couple. During the discharge process, the Zn metal anode undergoes an oxidation reaction, losing electrons to form Zn^2+^; the I_2_-containing cathode undergoes a reduction reaction, where part of I_2_ gains electrons and converts to I^−^. Meanwhile, I_2_ tends to further react with the generated I^−^, gradually forming soluble I_3_^−^, I_5_^−^, and other polyiodide intermediates. The generated intermediates further obtain electrons and convert them into I^−^. However, the dissolution and migration of these polyiodides between electrodes readily trigger the shuttle effect, which leads to active material loss and severe capacity decay. The reaction process can be summarized as follows^34^:

(4) $$Anode:Zn^{2+}+2e^{-}↔Zn$$

(5) $$Cathode:I_{2}+2e^{-}↔{2I}^{-}$$

(6) $$Overall:I_{2}+Zn↔Zn^{2+}+{2I}^{-}$$

Besides, it is important to note that Zn-S batteries operate through a multi-step solid-liquid-solid conversion, involving soluble polysulfide intermediates (e.g., S_4_^2−^, S_6_^2−^), whereas Zn-I_2_ batteries rely on a liquid-phase redox cascade centered on soluble polyiodides (I_3_^−^, I_5_^−^, and so forth).

Because of these mechanistic differences, their suitable application scenarios diverge. Zn-S batteries are better suited to static scenarios that pursue extreme energy density, whereas Zn-I_2_ batteries, benefiting from a liquid-phase reaction pathway and fast kinetics, fit high-power, frequently cycled dynamic applications. The following section will compare their practical application directions.

## Addressing the challenges: mechanisms and solutions

### Fundamental challenges

#### Sluggish kinetics

In contrast to Zn^2+^ ion intercalation reactions, which maintain the structural integrity of cathode materials, conversion-type cathodes undergo substantial structural transformations during electrochemical conversion processes. Specifically, the host structures are decomposed and subsequently reassembled, necessitating a substantial amount of energy. This energy requirement is significantly higher than that of intercalation reactions, where the host structure remains largely intact. Moreover, Zn^2+^ ion diffusion in conversion-type cathodes is severely hindered. The involvement of multiple electrons in conversion reactions creates a more complex electrochemical environment, while the strong interactions between Zn^2+^ ions and their surrounding lattice further restrict ionic mobility. Collectively, these factors lead to sluggish reaction kinetics, manifesting as limited rate capability, elevated overpotentials, and reduced power density. These drawbacks are particularly critical in applications requiring rapid charge/discharge rates or high-power performance.

#### Low conductivity

The inherently low electrical conductivity of S and I_2_ adversely affects redox reaction kinetics. During cycling, electron transport within S/I_2_-based cathodes cannot match the diffusion rate of Zn^2+^ ions in the electrolyte, resulting in incomplete utilization of active material sites and delayed redox processes. This imbalance between electronic and ionic transport directly constrains both the efficiency of active material use and the overall reaction kinetics. This challenge is especially prominent in Zn-S batteries. Beyond the low conductivity of elemental S, zinc sulfide (ZnS) as the primary discharge product of Zn-S batteries is also an electrical insulator. As the discharge progresses, ZnS accumulates on the cathode surface, forming a dense passivation layer. This layer not only impedes electron transfer between the active material and the current collector but also obstructs Zn^2+^ ion diffusion across the electrode-electrolyte interface. Consequently, Zn-S batteries exhibit significant voltage hysteresis and limited reversibility.

#### Low loading

Due to their thermodynamic instability and low conductivity, S and I_2_ typically require compositing with porous conductive carbonaceous materials or other matrix materials. These composites alleviate instability-induced material loss and improve electron transport, but the added inactive components occupy cathode space and increase overall cathode mass. This directly limits the mass proportion of sulfur and I_2_ in the cathode, resulting in active material loading far below the theoretical value needed for high energy density. Besides, the constrained loading is often paired with incomplete active material utilization, directly suppressing the energy density of ZIBs.

#### Shuttle effect

In Zn-S batteries, S_8_ undergoes stepwise reduction during discharge, generating soluble polysulfide intermediates such as S_4_^2−^ and S_6_^2−^, and finally converting to insoluble ZnS or S^2−^. During charging, ZnS is oxidized to S_8_, with soluble polysulfides also formed as intermediate species in the process. During the discharge of Zn-I_2_ batteries, I_2_ is reduced to polyiodide intermediates (I_3_^−^, I_5_^−^), which dissolve uncontrollably in the electrolyte and permeate the separator. During charging, these polyiodide ions can be oxidized back to solid I_2_; they undergo repeated oxidation-reduction cycles and shuttle between the two electrodes driven by concentration gradients.

The shuttle effect triggers a series of similar adverse consequences in both battery systems. It not only leads to continuous loss of active materials from the cathode but also induces self-discharge reactions at the Zn anode after polysulfide/polyiodide ions migrate to the anode side, resulting in Zn corrosion and surface passivation. Specifically, polyiodide ions are reduced to I^−^ at the Zn anode surface, while Zn is oxidized to Zn^2+^; polysulfide ions are progressively reduced to lower-order sulfides or S^2−^, accompanied by Zn oxidation. The resulting solid products (e.g., ZnS, ZnI_2_) continuously deposit on the anode, forming an insulating layer that aggravates Zn corrosion and irreversible active material consumption. This process not only significantly compromises the stability and cycle life of the Zn anode but also intensifies self-discharge and leads to deteriorating Coulombic efficiency. Furthermore, the shuttling of various dissolved intermediates causes localized concentration gradients in the electrolyte, further impairing charge-discharge efficiency and accelerating capacity fade, which collectively hinders the commercialization of aqueous Zn-S and Zn-I_2_ batteries.

Notably, Zn-S and Zn-I_2_ batteries differ systematically in terms of active species properties, reaction pathways, and side reaction mechanisms. In Zn-S batteries, the chain-like polysulfides exhibit complex morphologies and diverse valence states, involving multi-step redox processes with multiple sulfur valence transitions. These features lead to irreversible decomposition and active material loss during polysulfide migration, ultimately causing structural degradation and permanent capacity fading. In contrast, the I_2_ species in Zn-I_2_ batteries possess relatively simple and stable structures. Their reaction pathway centers on the highly reversible conversion between I^−^ and I_3_^−^, without involving multivalent changes, and the migrating species are theoretically recyclable. Nevertheless, this system still suffers from sluggish charge transfer kinetics and high energy barriers at the reaction interface, resulting in insufficient active material utilization and efficiency loss during practical cycling.

In short, Zn-S battery shuttling effect drives irreversible chemical degradation and permanent material loss, while Zn-I_2_ battery shuttling effect mainly leads to low efficiency and self-charge via reversible migration and charge-transfer limitations without fundamentally changing active-material morphology.

### Engineering strategies

#### Optimization of cathode host structure

The cathode materials in Zn-S and Zn-I_2_ batteries play a pivotal role in determining the performance and efficiency of the entire system, and researchers have focused on developing a variety of strategies to modify these cathodes.^35^ Due to the low conductivity of its material and the discharge product (ZnS ∼10^−9^ S cm^−1^), the utilization rate of active materials and reaction kinetics are severely restricted, which in turn limits the performance of Zn-S and Zn-I_2_ batteries.^36^^,^^37^ Moreover, pure I_2_ suffers from poor thermal stability, a direct consequence of its boiling point (184.3 °C).^38^ To tackle these problems, electrode modification is crucial, particularly for I-based cathodes, which must rely on host materials providing physical/chemical adsorption to simultaneously enhance thermal stability and conductivity. As a representative conductive host material, carbon leverages its high specific surface area and porous structure not only to significantly enhance electrode conductivity and ion transport efficiency, but also to encapsulate S and I_2_ species via physical adsorption and suppress their dissolution and shuttling.^39^^,^^40^ Various carbon materials can enhance the performance of batteries, such as graphite,^41^ carbon nanotubes (CNTs),^42^^,^^43^ MXenes,^44^^,^^45^ and the effectiveness varies depending on the type of carbon material used. Porous carbon is an ideal carbon-based host material for S and I_2_. In addition to the advantages of pore structure and specific surface area mentioned above, it also has a wide range of sources, mainly including metal-organic framework-derived porous carbon,^46^^,^^47^ organic matter-derived porous carbon,^48^ biomass-derived porous carbon,^49^^,^^50^ and so forth.

**Figure 2:**
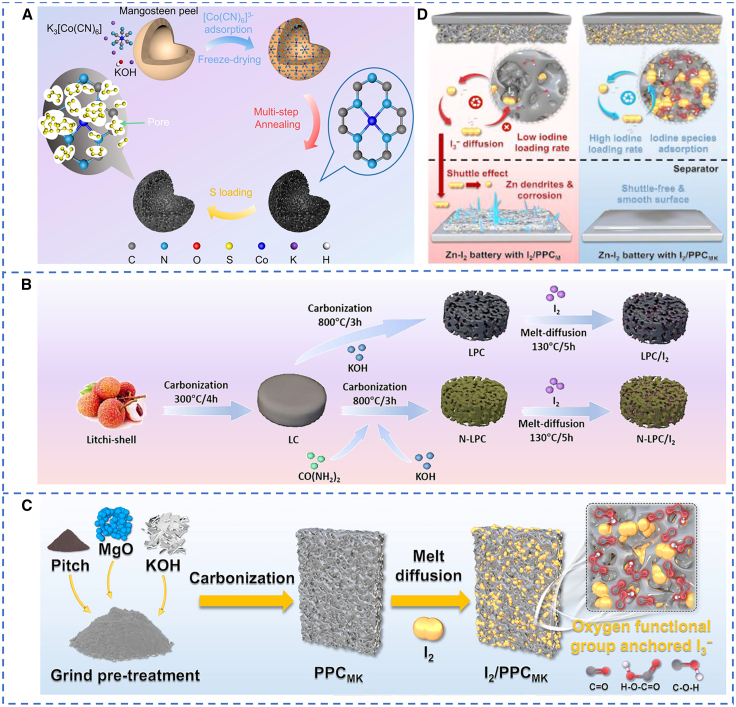
Schematic diagram of the preparation of a porous carbon cathode (A) Schematic representation of the preparation procedure of S@MMPC-Co. (A) reproduced with permission from Zhao et al.^49^ Copyright 2024 Elsevier. (B) Schematic illustration of the synthesis process of N-LPC/I_2_ and LPC/I_2_. (B) reproduced with permission from Ji et al.^50^ Copyright 2023 Elsevier. (C) Synthesis process for the I_2_/PPC_MK_. (D) The operational mechanism of I_2_/PP_CM_ and I_2_/PPC_MK_ as a cathode in Zn-I_2_ batteries. (C and D) reproduced with permission from Zeng et al.^53^ Copyright 2025 John Wiley and Sons.

In addition to poor conductivity, the volume expansion of discharge products is another critical challenge, resulting in mechanical failure of the cathode in Zn-S batteries.^35^ Porous carbon has great potential to alleviate the stress effect caused by the volume expansion of the sulfur cathode due to its good porosity.^51^ Zhao et al.^49^ synthesized micro-mesoporous carbon with single-atom cobalt (MMPC-Co) by a multistep heat treatment from mangosteen peel, which exhibited larger specific surface area, pore volume, and pore diameter, thereby providing more active sites (Figure 2A). The results reveal that the sulfur cathode (S@MMPC-Co) not only obviously increased electrical conductivity compared to pure sulfur, but also maintained good conversion-reaction kinetics and electrode structure integrity during the charge-discharge cycles. For Zn-S batteries, S@MMPC-Co retained a capacity of 729.96 mAh·g^−1^ after 500 cycles at a 4 A g^−1^ and achieved 507.21 mAh·g^−1^ at 5 A g^−1^ for high-rate capability (S loading: ∼4.33 mg cm^−2^). It is worth noting that doping carbon with heteroatoms such as N, O, or B strengthens carbon-I_2_ interactions, boosts the utilization of active materials, and effectively suppresses the shuttle effect.^48^^,^^50^^,^^52^ Ji et al.^50^ demonstrated a nitrogen-doped porous carbon derived from biomass litchi shell (N-LPC) as an I_2_ host for Zn-I_2_ batteries (Figure 2B). The highly porous framework enhances I_2_ loading and improves electronic and ionic transport, while nitrogen heteroatoms create plentiful anchoring sites that strengthen I_2_ binding and promote the reversible conversion between I_2_ and I^−^. The N-LPC/I_2_ cathode (I_2_ loading:∼1.5 mg cm^−2^) delivered a reversible capacity of 127 mAh·g^−1^ at 100 mA g^−1^, along with remarkable rate performance and cycling stability.

Research on heteroatom-doped carbon hosts for Zn-S batteries is relatively scarce and still in its early stages. Xu et al.^54^ used density functional theory (DFT) calculations to design nitrogen-doped carbon (NC) hosts and found that polysulfides are chemisorbed on N-containing sites, with adsorption energies between −3.23 and −3.98 eV. Gibbs free energy analyses further indicate that nitrogen doping facilitates polysulfide reduction. Experimental tests corroborated the theoretical predictions. NC/S cathodes outperform undoped amorphous carbon (AC)/S counterparts, providing a clear mechanistic basis and a useful strategy for rational cathode design.

In addition, oxygen-containing functional groups (─OH、─C─O、─COOH) on carbon host exhibit strong binding capacity with free I_3_^−^ in the electrolyte, which suppresses the dissolution of I_2_ species. This, in turn, enhances the performance of Zn-I_2_ batteries that operate via the single-step I^−^/I_2_ conversion reaction.^53^^,^^55^ Zeng et al.^53^ employed a one-step method to synthesize pitch-derived carbon (PPC_MK_) featuring micro-mesoporous structures and surface oxygen-containing functional groups (Figure 2C). The oxygen-containing functional groups of PPC_MK_ further enhance binding to I_3_^−^, improving polyiodide confinement (Figure 2D). Therefore, the Zn-I_2_ batteries delivered a high specific capacity of 236.76 mAh·g^−1^ (I_2_ loading: 4 mg cm^−2^) with an average Coulombic efficiency of 99.73% at 1 C, a low self-discharge (18.18% capacity loss after one week at rest), and excellent durability of 20 000 cycles at 20 C with 95.08% capacity retention. Nevertheless, studies on tailoring surface functionalities of carbon hosts in Zn-S batteries to strengthen chemisorption are still limited and require more in-depth exploration.

Moreover, rational design of the pore structure of carbon hosts is required to immobilize cathode active species via physical confinement. For Zn-S batteries, microporous carbons provide stronger sulfur confinement than mesoporous ones.^56^ In Zn-I_2_ batteries, pores below ∼1 nm effectively suppress the formation of I_3_^−^ and thus reduce polyiodide shuttle at its source.^57^ Pores in the 1–5 nm range can accommodate higher I_2_ loadings but are less effective at preventing I_3_^−^ formation, which weakens the adsorption of free I_3_^−^ and can lower Coulombic efficiency. Larger mesopores (>5 nm) and macropores exhibit even poorer adsorption of I_2_ species.^58^ Consequently, microporous carbons tightly anchor I_2_ molecules, whereas mesoporous carbons need to rely on chemical anchoring to restrain polyiodides. Overall, designing carbon hosts with tunable micro-mesoporous hierarchies is feasible for high I_2_ loading and strong immobilization. At the same time, combining multiple adsorption mechanisms and tailoring the porosity distribution, surface chemistry, and conductivity of electrodes to the cathode’s properties is necessary to maximize performance.

Therefore, optimization strategies for carbon hosts, including heteroatom doping, surface functionalization, and pore structure regulation, have been demonstrated to collectively mitigate issues of low conductivity and shuttle effects in Zn-S and Zn-I_2_ batteries. Nevertheless, these approaches still face a fundamental trade-off between high mass loading and robust anchoring capability, alongside challenges in the scalable synthesis of certain complex carbon architectures, which hinder their practical deployment.

#### Electrocatalytic boost

The precise enhancement of cathode catalytic activity is regarded as the “third electrode” driving force for high areal capacity and long cycle life in Zn-S and Zn-I_2_ batteries. Its goal is to construct on a conductive scaffold an interface that is both highly active and intrinsically stable, capable of accelerating multi-electron conversions, anchoring intermediate species, and accommodating volume changes. The following is mainly about carbon doped with heteroatoms, single metal atoms, and alloyed nanoclusters, and respectively explains how it lowers reaction barriers, suppresses the shuttle effect, and induces reversible deposition in Zn-S and Zn-I_2_ batteries, thereby upgrading S and I_2_ cathodes from insulating hosts to autocatalytic electrodes.

**Figure 3:**
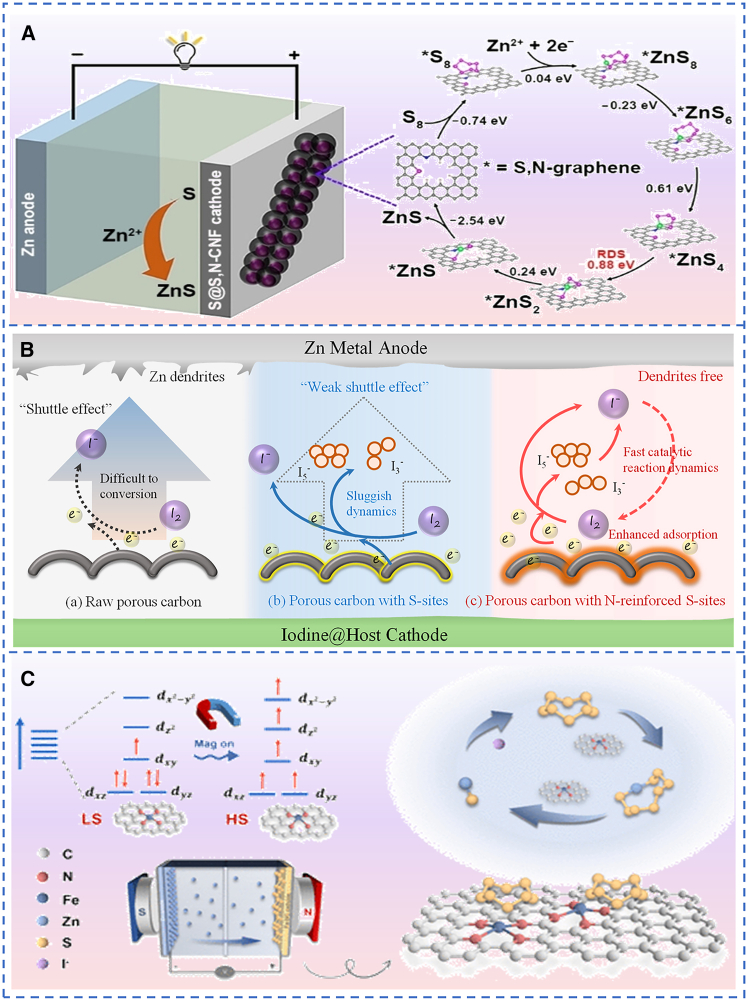
Schematic diagram of the preparation of the catalyst-doped carbon host (A) Schematic diagram of the electrocatalytic SRR process on S@S,N-CNF in aqueous Zn-S batteries. (A) reproduced with permission from Li et al.^59^ Copyright 2024 John Wiley and Sons. (B) Mechanism diagram of iodine conversion reaction in Zn-I_2_ batteries with different porous carbon hosts. (B) reproduced with permission from Feng et al.^60^ Copyright 2024 Elsevier. (C) Schematic illustration of the transformation of the S@Fe-NC electrode in a Zn-S battery under an applied magnetic field and the electrocatalytic SRR process within the battery. (C) reproduced with permission from Dai et al.^61^ Copyright 2025 American Chemical Society.

Heteroatom-doped carbon hosts provide a catalytic platform for S and I_2_ redox reactions by redistributing charge and offering tunable local coordination environments. In Zn-S batteries, carbon nanofibers doped with both sulfur and nitrogen (N) serve as the host. S and N dopants cooperatively catalyze the sulfur reduction reaction by lowering energy barriers and accelerating kinetics, which raises the discharge voltage and increases specific capacity. DFT calculations further indicate that dual S and N doping lowers both thermodynamic and kinetic barriers for the conversion of ZnS_4_ to ZnS_2_, thereby markedly accelerating sulfur reduction kinetics as shown in Figure 3A.^59^ Compared with the Zn-S battery system, research on heteroatom-doped carbon-based Zn-I_2_ batteries is currently more extensive, and the mechanistic roles of doped-atoms in regulating the iodine redox reaction have been more systematically elucidated. Applying the same strategy to the Zn-I_2_ batteries, a nitrogen-sulfur double-heteroatom doped carbon material functions as an effective iodine host.^60^ N-doping enhances the adsorption of iodine species on the carbon matrix, but the catalytic promotion of iodine conversion is limited. S-doping facilitates charge transfer and reduces the conversion barriers of I_3_^−^ and I_5_^−^, producing substantially improved catalytic kinetics (Figure 3B). The synergistic effect of S and N improves chemical adsorption and catalytic activity for iodine conversion and enables stable cycling for 15 000 cycles at 50°C.

Transition metal single-atom catalysts (M-N_4_, M = Co, Fe, Cu, and so forth) offer “one-to-one” electron transfer pathways for sulfur/iodine species, featuring 100% atomic utilization efficiency and tunable d-band centers.^62^^,^^63^^,^^64^^,^^65^ To fully exploit their potential in Zn-S batteries, iron-based single-atom catalysts (Fe-SAs) have been integrated with external magnetic field modulation.^61^ Under this magnetic field, Fe-SAs undergo a spin-state transition from low-spin to high-spin, which optimizes the adsorption of intermediates, accelerates electron transfer, and thereby synergistically enhances the sulfur conversion capability of Fe-SAs (Figure 3C). The results show that under the influence of the magnetic field, the sulfur cathode with high-spin Fe-SAs catalysts achieved a specific capacity of 1399 mAh·g^−1^ at 5.0 A g^−1^ and exhibited excellent cycling stability over 300 cycles. Shifting to Zn-I_2_ batteries, Yang et al. confirmed that single-atom catalysts (SACs), including SACu, SACo, SANi, SAFe, SAMn, SAV, SAZn, and SATi, which are used for the adsorption and conversion of I_3_^−^, thus suppressing the shuttle effect of I_3_^−^. The selection of metal active centers in SACs must not only meet the requirements of catalytic activity, but also possess good reversibility. They should maintain stable catalytic activity without poisoning after interacting with different forms of I_2_ substances, thereby ensuring the continuous stability of the catalytic process. SACu exhibits the best catalytic activity and reasonable interactions with different I_2_ substances, which can effectively avoid the shuttle effect of I_3_^−^ and maximize the utilization efficiency of iodine active materials. It can be applied in the electrode design of high-performance rechargeable batteries.^66^

Alloyed nanoclusters (Cu_x_Co_1−x_, FeNi, PtCu_3_) leverage the d-band coupling and strain effects from multi-metal synergy to construct “breathable” catalytic-confinement microreactors on the surface of carbon skeletons. In Zn-S batteries, Zhang et al.^67^ prepared high-capacity cathodes via *in-situ* interfacial polymerization of Fe(CN)_6_^4−^-doped polyaniline within sulfur nanoparticles. The functional polymer, acting as a Zn^2+^ cation carrier, enables rapid cation transport. During discharge, Zn_x_Feᴵᴵᴵ(CN)_6_ and sulfur undergo synchronous reduction to form ZnᵧFeᴵᴵ(CN)_6_ and ZnS. The pre-encapsulated Zn^2+^ within the open channels of Fe(CN)_6_n^−^ spontaneously integrates with sulfur (S_8_ + Zn_2_Feᴵᴵ(CN)_6_ ↔ ZnS + Zn_1.5_Feᴵᴵᴵ(CN)_6_, ΔG = −24.7 kJ mol^−1^), accelerating the sulfur reduction kinetics. During charging, the iron redox active centers efficiently catalyze ZnS oxidation at low overpotentials. The cathode with a sulfur content of 70 wt % delivers a reversible capacity of 1205 mAh·g^−1^, an operating voltage plateau of 0.58 V, a decay rate of 0.23% per cycle over 200 cycles, and an energy density of 720 Wh·kg^−1^.

In Zn-I_2_ batteries, iron nitride nanoclusters dispersed in a three-dimensional porous carbon framework (PC@Fe_2_N) enable efficient iodine enrichment and catalysis of its reversible conversion. Orbital hybridization occurs between Fe_2_N and iodine on the carbon substrate, optimizing the electronic structure and reducing reaction energy barriers. This promotes polyiodide conversion, endowing the battery with both excellent rate performance and an ultra-long cycle life of 20,000 cycles.^68^

In summary, electrocatalytic enhancement strategies have demonstrated remarkable effectiveness in accelerating redox kinetics, suppressing shuttle effects, and improving active material utilization in Zn-S and Zn-I_2_ batteries. Nevertheless, their practical deployment is hindered by several critical limitations: the challenge of precisely controlling catalytic active sites, inherent trade-offs between catalytic activity and long-term stability, and the increased complexity of synthesis processes that may impede large-scale commercialization.

#### Optimization of electrolyte formulation

As the “liquid bridge” connecting the two electrodes, the electrolyte’s formulation design directly regulates the active sites at the cathode interface, ion flux, and reaction pathways, which represent another key to enhancing capacity and cycle life.^69^ Through the synergistic regulation of “ions-interface-solvent,” the electrolyte simultaneously reduces the energy barrier for sulfur/iodine conversion, anchors shuttling species, and suppresses side reactions. This provides a highly reversible and low-polarization cathode for Zn-S and Zn-I_2_ batteries.

Direct replacement of weakly coordinating zinc salts can reshape solvation chemistry at the ion pair level, release free Zn^2+^ ions, and simultaneously reduce the bond dissociation energy barrier of sulfur/iodine, thereby activating the redox conversion of sulfur and iodine cathodes. In Zn-S batteries, substituting traditional ZnSO_4_ electrolytes with Zn(OTf)_2_,^70^ Zn(TFSI)_2_,^71^ or ZnCl_2_^72^ significantly weakens the coordination between Zn^2+^ and H_2_O. This effectively suppresses the side reaction of H^+^ co-deposition on the cathode side, while releasing more free Zn^2+^ ions to promote the formation of stable complexes with S_8_ or S_n_^2−^. These changes reduce the overpotential of the two-electron reduction reaction of sulfur and alleviate the deposition of insulating ZnS passivation layers, thereby improving the battery’s cycling stability and reversible capacity. Taking Zn(OTf)_2_ as an example, it not only achieves a reversible capacity of 788 mAh·g^−1^ but also exhibits higher capacity retention due to its higher Zn nucleation overpotential and slower corrosion reactions.^70^ When this strategy is applied to Zn-I_2_ batteries, weakly coordinating Zn(OTf)_2_ can also precisely regulate the I^−^/I_3_^−^/I_2_ redox pathways. Zhang et al.^73^ proposed the cathode/electrolyte mutualistic aqueous (CEMA) mechanism based on the oxidizing ability of OTf^−^ toward I_3_^−^. Nanoporous carbon current collectors spontaneously adsorb and enrich I^−^/I_5_^−^ in aqueous Zn(OTf)_2_, forming iodine cathodes with high areal capacity. While OTf^−^ weakens the Zn^2+^-H_2_O coordination, it also reduces the charge transfer resistance of I_5_^−^/I^−^ and suppresses the competitive hydrogen evolution reaction. As a result, the CEMA Zn-I_2_ battery maintains 76.9% of its capacity after 1000 cycles at 0.5 mA cm^−2^, and still retains 74.6% of its capacity at a rate of 5 mA cm^−2^.

**Figure 4:**
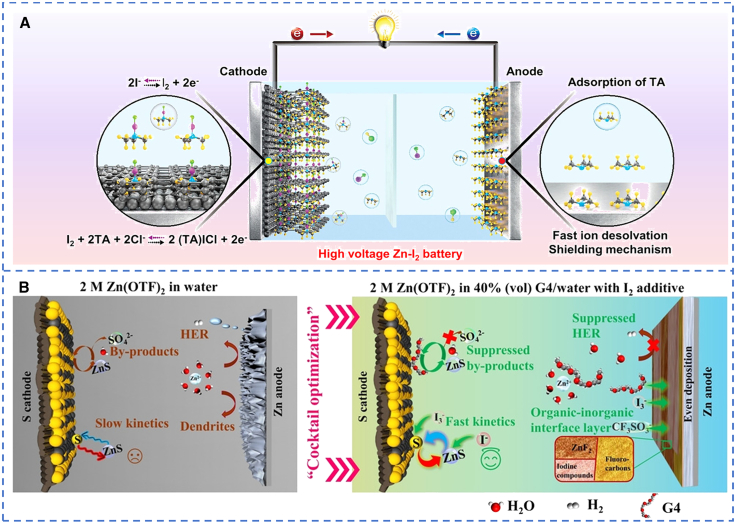
Schematic diagram of the preparation of an efficient electrolyte (A) Schematic illustration of the mechanism of high-voltage aqueous Zn-I_2_ batteries with electrolyte additive TAH. (A) reproduced with permission from Wu et al.^79^ Copyright 2024 John Wiley and Sons. (B) Schematic illustration of aqueous Zn–S batteries using (up) 2M Zn(OTF)_2_ in water electrolyte and (down) 2M Zn(OTF)_2_ in 40% (vol) G4/water with I_2_ additive. (B) reproduced with permission from Hei et al.^82^ Copyright 2022 John Wiley and Sons.

Functional additives leverage the dual-functional interfacial effect of “catalysis-anchoring” to accelerate electron transfer and block the escape of polysulfides/polyiodides. They have emerged as an effective approach to simultaneously suppress sulfur and iodine shuttling.^74^^,^^75^^,^^76^ For Zn-S batteries, redox mediators such as thiourea form soluble Zn-S intermediate coordination clusters in the liquid phase.^77^ This reduces the energy barrier for S_8_ ring cleavage and regulates the deposition morphology of ZnS from insulating particles to a conductive network. Iodine-based additives can significantly promote ZnS oxidation. Li et al.^78^ proposed that I_2_ serves both as a Zn^2+^ transfer medium and a catalyst for sulfur conversion. During discharge, I_2_ is first reduced to I^−^, which then coordinates to form I_3_^−^. During charging, ZnS and I_3_^−^ undergo synchronous oxidation. The excellent reversibility of the I_2_↔ I_3_^−^ couple allows I_2_ to remain stable and act as a Zn^2+^ carrier. With the addition of I_2_, the S@CNTs cathode achieves a high capacity of 1105 mAh·g^−1^ and an energy density of 502 Wh·kg^−1^. Quaternary ammonium cations simultaneously suppress Zn dendrite growth and the cross-diffusion of sulfur species by electrostatically shielding the anode. Tetraalkylammonium iodide (R_4_NI) enables a solid-liquid-solid reaction pathway through a dual-medium innovation.^79^ During discharge, R_4_N^+^ induces the formation of soluble polysulfides (S_4_^2−^/S_6_^2−^), which rapidly combine with Zn^2+^ to form ZnS. This converts the sulfur cathode reaction from “solid-solid conversion (S-ZnS)” to “solid-liquid-solid conversion (S-polysulfides-ZnS),” greatly promoting reaction kinetics. Meanwhile, R_4_N^+^ limits the solubility of I_3_^−^, overcoming the shuttle effect caused by anode corrosion and catalyst consumption, and ultimately reducing the charge-discharge polarization significantly. Turning to Zn-I_2_ batteries, trimethylammonium chloride (TAH), a multifunctional inorganic salt electrolyte additive, enables a stable high-valent iodine cathode with high theoretical specific capacity in the four-electron transfer I^−^/I_2_/I^+^ reaction. This is achieved through the unique bidentate coordination structure between the amine group (TA) and ICl.^80^ The TAH additive not only activates high-valent iodine and reduces the desolvation energy barrier to mitigate concentration polarization (Figure 4A) but also enables dense and dendrite-free Zn deposition. This allows the Zn anode to cycle stably with a maximum areal capacity of 57 mAh·cm^−2^ and a Zn utilization rate of up to 97%. For the dual-functional Zn pyrrolidone carboxylate additive, its anion preferentially coordinates with I_2_ via Lewis acid-base interactions. This reduces the concentration of polyiodides to suppress shuttling and optimizes iodine conversion kinetics. At the same time, it adsorbs on the Zn anode to inhibit Zn corrosion and promote non-dendritic deposition. The Zn-I_2_ full cell assembled based on this additive achieves a high specific capacity of 211 mAh·g^−1^ (≈100% iodine utilization) and retains 87% of its capacity after more than 30,000 cycles.^81^

The introduction of advanced cosolvent systems can widen the electrochemical window while regulating the solubility of sulfur/iodine. This provides a reaction environment with low polarization and high stability for the reversible conversion of sulfur/iodine, and creates an ideal microenvironment for the cathode with low water activity and matched dielectric constant.^82^^,^^83^ In Zn-S batteries, a “cocktail-optimized” electrolyte with cosolvent-catalyst synergy is constructed using zinc trifluoromethanesulfonate as the zinc salt, tetraethylene glycol dimethyl ether (G4) and water as cosolvents, and iodine as the additive (Figure 4B). This electrolyte not only activates efficient I_3_^−^/I^−^ catalysis but also enhances the wettability of the electrolyte on the sulfur cathode and blocks the access of water. These effects improve sulfur conversion kinetics and suppress the formation of SO_4_^2-^-based byproducts. Additionally, it helps form a stable organic-inorganic solid electrolyte interphase (SEI) on the surface of the Zn anode. The battery maintained 70% of its capacity after 600 cycles at 4 A g^−1^.^84^ For Zn-I_2_ batteries, an acidic deep eutectic solvent (ZPDES) prepared with H_3_PO_4_ and ZnCl_2_ can suppress corrosion caused by protons and Cl^−^, accelerate I^−^/I^0^/I^+^ redox kinetics, and guide Zn (002) deposition. These features support the four-electron redox reaction in Zn-I_2_ batteries, which retained a high specific capacity of 576 mAh·g^−1^ after 320 cycles at 0.5 A g^−1^ and achieved 100% capacity retention over 20,000 cycles at 7 A g^−1^.^85^

The construction of hydrogel electrolytes or high-concentration electrolytes is another effective approach to suppress the shuttle effect. In Zn-I_2_ batteries, a cation-conduction dominated hydrogel (CCH) with anions (─SO_3_^−^) bonded via chemical linkages exhibits a Zn^2+^ transference number of 0.81, which is more than twice that of the dual-ion conductive polyacrylamide hydrogel with ZnSO_4_ (DICH). Through the interaction between sulfonate groups and Zn, it promotes the dense deposition of Zn (002), optimizes Zn^2+^ solvation, and mitigates corrosion. Meanwhile, amide groups facilitate Zn^2+^ transport. When applied in Zn-I_2_ cells, the ─SO_3_^−^ groups electrostatically repel polyiodides to suppress shuttling, resulting in a capacity decay rate of 0.0076% per cycle over 22,000 cycles at 10 C.^86^ In addition, a water-in-salt (WIS) electrolyte prepared with high-concentration ZnCl_2_ and KI can inhibit the formation and diffusion of I_3_^−^. In the WIS electrolyte, the average number of water molecules solvating each iodide ion is much lower than that in traditional aqueous electrolytes. The strong coordination between water and Zn^2+^ cations drastically reduces water chemical activity. Combined with the strong adsorption of the microporous structure of the reduced graphene oxide (rGO) host, iodine species are significantly confined within the rGO electrode, thereby suppressing the shuttle effect. This battery delivers an ultra-high capacity of 6.5 mAh·cm^−2^ at 2 mA cm^−2^ and a substantially improved Coulombic efficiency of 95%. After 2000 cycles, it still maintains excellent cycling stability with a capacity of 2 mAh·cm^−2^ at 50 mA cm^−2^, nearly retaining 100% of its capacity.^87^

In conclusion, electrolyte optimization is vital for Zn-S and Zn-I_2_ batteries to regulate reaction pathways and suppress shuttling and side reactions, thereby boosting reversibility and stability. Despite its importance, these strategies suffer from limitations such as complex design, electrode compatibility issues, and increased cost or reduced ion transport efficiency in some high-performance systems.

#### Separator modification

Functional separators are evolving from “passive isolation” to “active regulation.” Through the triple design of pore charge, surface chemistry, and gradient structure, they simultaneously block the shuttling of polysulfides/polyiodides, regulate Zn ion transport, and act as secondary reaction interfaces. This significantly enhances the cycling stability and safety performance of Zn-S and Zn-I_2_ batteries. However, research on separator modification for aqueous Zn-S batteries is still limited, so the following discussion focuses on functional separators in Zn-I_2_ battery systems.

The ion sieving-electrostatic repulsion strategy leverages pore size matching and charge repulsion to confine intermediate product anions to the cathode side while allowing the transport of Zn^2+^. In Zn-I_2_ batteries, metal-organic frameworks (MOFs) used as ion sieve membranes suppress the shuttling of I_3_^−^ and mitigate harmful side reactions on Zn anodes. Additionally, by regulating the solvation structure of the electrolyte, MOF channels construct a unique electrolyte structure with a higher degree of aggregated ion association than saturated electrolytes. After the introduction of this multifunctional MOF membrane, the performance of both iodine cathodes and Zn anodes is improved simultaneously. Ultimately, the Zn-I_2_ battery achieves an ultra-long cycle life (>6000 cycles), high capacity retention (84.6%), and high reversibility (CE: 99.65%).^88^

Introducing polar or catalytic sites on the separator surface traps the dissolved intermediate products and rapidly converts them into the next-stage products, reducing the shuttling amount. In Zn-I_2_ batteries, glass fiber (GF) is modified using N/O co-doped CNTs with Fe and Ni as dual catalysts to develop a novel separator (CNTs@CW/GF). The synergistic interaction between the three-dimensional porous carbon framework and heteroatom doping not only provides a physical confinement effect but also offers multiple anchoring sites. These features promote the chemical adsorption of iodine and accelerate I_2_/I^−^ redox kinetics to mitigate the shuttle effect. Additionally, the CNTs exhibit high electrical conductivity, continuous charge transport pathways, and structural stability. More importantly, the Fe-Ni alloy encapsulated in the CNTs can act as catalytic centers, enhancing polyiodide redox kinetics through catalytic conversion and effectively improving iodine conversion efficiency. Consequently, the Zn|CNTs@CW/GF|I_2_-AC battery delivers a high discharge capacity (170.4 mAh·g^−1^ at 0.1 A g^−1^) and excellent rate performance (82.7 mAh·g^−1^ at 10 A g^−1^), along with reduced polarization, accelerated reaction kinetics, and improved I_2_ conversion efficiency.^89^

Asymmetric separator design provides an effective approach to addressing this challenge to a certain extent through selective ion sieving, enabling specific interception on the cathode side and uniform Zn deposition on the anode side. Zhang et al. designed a nanocellulose-mediated, cation-selective, multi-scale ion-sieving heterostructured separator. This separator can simultaneously inhibit anion migration and homogenize the electric field distribution at the separator/electrode interface. It also maintains a nanoscale uniform Zn^2+^ concentration across the entire electrode surface, thereby achieving highly reversible and dense Zn deposition. Corrosion and side reactions during cycling in corrosive electrolytes are negligible. These advantages endow Zn-I_2_ batteries with cathodes featuring a high I_2_ mass loading of 13.0 mg cm^−2^ with remarkable reversibility, delivering a discharge capacity of 172.1 mAh·g^−1^ after 1000 cycles.^90^

Functional separator strategies for Zn-I_2_ batteries effectively suppress polyiodide shuttling, inhibit Zn dendrite formation, and promote redox kinetics. Nevertheless, their practical implementation is hindered by elevated fabrication complexity, increased material costs, and the persistent challenge of balancing rapid Zn^2+^ transport against efficient polyiodide interception.

#### Multi-electron reactions strategy

Facilitating a more efficient multi-electron transfer process represents a promising strategy to enhance the battery’s specific capacity, which not only improves active material utilization but also mitigates capacity fading over cycles. In Zn-S batteries, the two-step four-electron conversion (S ↔ CuS ↔ Cu_2_S) of S-Cu cathodes suffers from significant kinetic bottlenecks. The preferential oxidation of Cu^+^ to Cu^2+^ tends to form intermediate CuS, while the irreversible oxidation of CuS → S causes active material loss and capacity decay. Meanwhile, the high ion/electron transport resistance during solid-solid phase transitions leads to potential hysteresis. To address these challenges, Zhang et al. proposed a “spatial confinement” strategy that leverages the physical confinement effect of a carbon matrix to redirect the reaction pathway from the preferential oxidation of Cu^+^→Cu^2+^ (leading to CuS formation) toward the oxidation of S^2−^→S^0^.^91^ This not only suppresses the formation and irreversible conversion of CuS but also reduces the mass transfer resistance during phase transitions, ultimately enabling a highly reversible one-step four-electron conversion of Cu_2_S → S. The resulting Se_4_S_4_/spatial confinement-carbon composite exhibited excellent cycling stability in half-cells, sustaining the four-electron reaction stably.

The multi-valent nature of iodine unlocks new redox pathways in Zn-I_2_ batteries, resulting in a greater electron transfer number and a higher reaction potential to achieve superior energy density.^92^^,^^93^ A common strategy employs halogen ion (Cl^−^/Br^−^) additives that react with I_2_ to form ICl/IBr interhalogen compounds, thereby stabilizing I^+^ species and triggering sequential I^−^/I_0_/I^+^ electron transfer to unlock high-valence iodine redox. For example, Zou et al. employed ZnCl_2_ electrolyte, leveraging I^+^/I_2_ (1.83 V vs. Zn^2+^/Zn) and I_2_/I^−^ (1.29 V vs. Zn^2+^/Zn) redox couples to deliver 750 Wh·kg^−1^ energy density, and stable cycling over 6,000 cycles.^94^ Besides, halogen interchange chemistry mediated by Br^−^ further unlocks a six-electron IO_3_^−^/I^−^ redox reaction, achieving an exceptional energy density of 1,357 Wh·kg^−1^.^95^ The cycling process centers on halogen interchange between electrode I_2_ and electrolytic Br^−^: charging involves nucleophilic attack by H_2_O on the polar IBr intermediate to generate IO_3_^−^, while discharging features Br^−^-catalyzed dissociation and reduction of IO_3_^−^ to IBr and Br_2_. More impressively, as a cost-effective electrode binder, commercially available polyacrylamide (PAM) powder was demonstrated to catalyze the reversible conversion between I_0_ and I^+^, enabling a stable four-electron transfer reaction in the absence of halogen ions.^96^ Research shows that PAM from various vendors functions as a catalytic binder, where abundant nucleophilic -CONH_2_ groups trigger the four-electron process through strong binding with I^+^. Table 2 further summarizes the recent advances in Zn-I_2_ batteries enabled by alternative multi-electron conversion mechanisms. While the multi-electron reaction strategy imparts significant potential for high-energy-density and rapid kinetic performance to Zn-I_2_ batteries, its practical implementation presents notable challenges. The same reactions that contribute to high capacity also give rise to complex side effects, such as the shuttling of intermediate species, highlighting that this promising approach remains an area of active research requiring further systematic investigation.

**Table 2:** Comparisons of the electrochemical performances of multi-electron reaction based Zn-I_2_ batteries

Cathode Structure	Electrolyte	Energy density (Wh·kg^−1^)	Capacity	Cycling Life (cycles)	Reference
I_2_/C	2M ZnSO_4_ + 0.5M TAH	–	450 mAh·g^−1^ at 2 A g^−1^	5000	Wang et al.^80^
PAC-I_2_	1M ZnSO_4_	750 Wh·kg^−1^	609 mAh·g^−1^ at 0.2 A g^−1^	6000	Zou et al.^94^
I_2_/HAC	0.1M H_2_SO_4_ + 0.1M KBr	1357 Wh·kg^−1^	950 mAh·g^−1^ at 1 A g^−1^	150	Ma et al.^95^
Fe_SA_-NC/I_2_	2m ZnCl_2_ (PEG)	723 Wh·kg^−1^	521 mAh·g^−1^ at 1 A g^−1^	20000	Liu et al.^97^
Fe_SA_-NC/I_2_	ZnCl_2_, LiCl, and acetonitrile	420 Wh·kg^−1^	653.8 mAh·g^−1^ at 1 A g^−1^	1000	Liu et al.^98^
pMA@AC/I_2_	2M ZnSO_4_	–	405 mAh·g^−1^ at 3.9 A g^−1^	10000	Wang et al.^99^
AC/I_2_	30m ZnCl_2_	–	582 mAh·g^−1^ at 1 A g^−1^	20000	Yan et al.^100^
C_3_H_9_IS/TZ-COFs	Zn(OTF)_2_-MPIBr	1464 Wh·kg^−1^	1296 mAh·g^−1^ at 1 A g^−1^	1200	Du et al.^101^

## Summary and perspectives

### Summary

Zn-S and Zn-I_2_ batteries, as representative ZIBs based on conversion-type cathodes, have emerged as strong contenders for next-generation energy storage due to their high theoretical energy density, abundant resources, and environmental friendliness. However, these battery systems still face a series of significant challenges in practical industrial applications.

#### Unclear reaction mechanisms

The sulfur reduction pathway in Zn-S batteries remains hotly debated: some studies advocate a direct S-to-ZnS conversion, whereas others invoke stepwise redox via soluble polysulfide intermediates. This fundamental scientific issue requires further clarification by combining advanced *in situ* spectroscopic techniques with theoretical calculations. Meanwhile, although Zn-I_2_ batteries are characterized by highly reversible I^−^/I_3_^−^ conversion reactions, the microscopic mechanisms of interfacial charge transfer and dynamic regulation strategies for solvation structures still need in-depth exploration. Breakthroughs in these foundational scientific questions will provide important guidance for further performance improvements.

#### Kinetic and conductivity limitations

Both battery systems are severely constrained by sluggish conversion reaction kinetics and the intrinsically low conductivity of active materials. In Zn-S batteries, the continuous accumulation of insulating ZnS products and significant volume expansion during charge/discharge cycles critically affect electrode structural stability. In Zn-I_2_ batteries, the slow diffusion of iodine species directly limits reaction rates. These factors collectively lead to poor rate performance and low active material utilization, representing key technical bottlenecks hindering practical application.

#### Shuttle effect and side reactions

The dissolution and migration of polysulfides and polyiodides in the electrolyte trigger severe shuttle effects. This phenomenon not only causes continuous loss of cathode active materials but also leads to Zn anode corrosion, hydrogen evolution reactions, and battery self-discharge. The synergistic effect of these side reactions significantly reduces Coulombic efficiency and cycle life, constituting a core issue affecting long-term battery stability.

#### Poor compatibility between the electrolyte and the electrode

Traditional aqueous electrolytes demonstrate limited transport efficiency for multivalent ions. Although high-concentration electrolytes offer benefits in mitigating side reactions, they typically result in a substantial increase in viscosity, which compromises ionic conductivity. Moreover, inadequate electrode-electrolyte interfacial stability and the absence of a systematic co-design approach hinder the simultaneous realization of high active material loading and high interfacial reversibility, thereby significantly constraining further enhancements in energy density.

### Prospects and key research directions

To advance Zn-S and Zn-I_2_ batteries from laboratory research to practical applications, future efforts should focus on the following key directions.

#### Mechanism elucidation and theoretical guidance

There is an urgent need to develop multi-scale *in situ* characterization techniques (e.g., *in situ* X-ray absorption spectroscopy, Raman, cryo-electron microscopy) to enable real-time dynamic tracking of active material phase transitions and interface evolution during charge/discharge. Concurrently, combining machine learning-assisted molecular dynamics simulations to establish complete structure–activity relationships from “electrolyte composition-interface structure-battery performance” will provide a solid theoretical basis and design guidelines for new material design and electrolyte system optimization.

#### Cathode structure engineering and catalytic design

In developing multifunctional host materials, it is essential to construct composite structures integrating polar sites (e.g., MoS_2_, MXene) with three-dimensional conductive networks (e.g., N-doped carbon) to achieve synergistic effects of efficient anchoring and catalytic conversion of polysulfides/polyiodides. The introduction of single-atom catalysts will be an important research direction. Precise design of metal active centers (e.g., Fe, Co) can effectively regulate sulfur/iodine reaction pathways, significantly reduce reaction energy barriers, and enhance the reversibility of conversion reactions. Besides, gradient structured cathodes use inner high-conductivity carbon frameworks for ion/electron transport and outer polar catalytic sites for polysulfide/polyiodide anchoring. This eases volume expansion and optimizes stress distribution. Heterogeneous structures combine flexible buffer components such as elastic polymer matrices with rigid conductive skeletons. They suppress electrode cracking from volume changes and maintain continuous conductivity, boosting long-cycle reliability.

#### Electrolyte engineering and interface regulation

The forthcoming research agenda must prioritize the development of electrolyte systems that fulfill dual functions within a single phase. Molecular designs exhibiting high selectivity for Zn ion coordination can simultaneously regulate Zn nucleation and deposition at the anode while reducing the activation barrier for sulfur or iodine redox conversion at the cathode, thereby integrating deposition control with reaction kinetics enhancement in a unified medium. Concurrently, efforts should focus on gel architectures featuring polymer backbones dynamically cross-linked via reversible boronic ester bonds or analogous motifs. These networks effectively suppress solvent evaporation, accommodate mechanical deformation, and maintain stable interfacial contact across a wide temperature range making them particularly suitable for wearable and flexible electronic devices. Beyond liquid-based systems, solid-state batteries employing non-volatile electrolytes in the form of dense ceramic or polymer-ceramic composite membranes provide a hermetically sealed, leak-free environment. Such configurations isolate metallic Zn from ambient moisture and oxygen, homogenize ion flux across electrode interfaces, and significantly extend cycle life and tolerance to mechanical and thermal abuse.

#### New material systems and cross-technology integration

Exploring asymmetric electrolyte configurations will be an innovative direction. By employing highly viscous gel electrolytes on the cathode side and flowing electrolyte systems on the anode side, precise regulation of different functional zones can be achieved. Furthermore, actively leveraging advanced catalytic medium design concepts from lithium-sulfur batteries (e.g., solid-phase conversion promoters) will promote the application breakthroughs of Zn-S/Zn-I_2_ batteries under extreme environmental conditions, providing new solutions for energy supply in special scenarios.

## Acknowledgments

The research was supported by the 10.13039/501100001809National Science Foundation of China (10.13039/501100001809NSFC No. 52405192) and the 10.13039/501100004608Natural Science Foundation of Jiangsu Province (BK20210480, BK20221104).

## Declaration of interests

The authors declare no conflicts of interest for this work.

## References

[1] Huang J., Wang Z., Hou M., Dong X., Liu Y., Wang Y., Xia Y. (2018). Polyaniline-intercalated manganese dioxide nanolayers as a high-performance cathode material for an aqueous zinc-ion battery. Nat. Commun..

[2] Park J.-H., Choi J.H., Seo J.-W., Kim I., Nam J.S., Kim J.-H., Jin H.M., Choi S.-J., Oh P., Jung J.-W. (2025). Minuscule ZnV_2_O_4_ entrapped carbon nanofiber composite cathode for long-lasting aqueous Zn-ion batteries. Adv. Fiber Mater..

[3] Grignon E., Battaglia A.M., Schon T.B., Seferos D.S. (2022). Aqueous zinc batteries: Design principles toward organic cathodes for grid applications. iScience.

[4] Liu Y., Hu Q., Zhong J., Wang Z., Guo H., Yan G., Li X., Peng W., Wang J. (2020). A renewable sedimentary slurry battery: preliminary study in zinc electrodes. iScience.

[5] Jia H., Liu K., Lam Y., Tawiah B., Xin J.H., Nie W., Jiang S.-x. (2023). Fiber-based materials for aqueous zinc ion batteries. Adv. Fiber Mater..

[6] Qu X., Xie H., Li N., Wang P., An X., Zhang W., Zhao Z., Shi X. (2025). Energizing sulfur chemistry: Synergistic modulation of oxygen vacancies and heterointerface in MoO_2_-x-Mo_2_C@ NC for long-lasting lithium-sulfur batteries. Nano Research Energy.

[7] Kang J., Zhao Z., Li H., Meng Y., Hu B., Lu H. (2022). An overview of aqueous zinc-ion batteries based on conversion-type cathodes. Energy Mater..

[8] Xu H., Yang W., Li M., Liu H., Gong S., Zhao F., Li C., Qi J., Wang H., Peng W., Liu J. (2024). Advances in aqueous zinc ion batteries based on conversion mechanism: challenges, strategies, and prospects. Small.

[9] Wang H., Chen S., Fu C., Ding Y., Liu G., Cao Y., Chen Z. (2021). Recent advances in conversion-type electrode materials for post lithium-ion batteries. ACS Mater. Lett..

[10] Wang K., Min S., Wu Q., Jia H. (2024). Preparation and electrochemical performance of a flexible MoS_2_ electrode based on carbon fiber fabric. Basic Sci. J. Text. Univ..

[11] Guo Y., Chua R., Chen Y., Cai Y., Tang E.J.J., Lim J.J.N., Tran T.H., Verma V., Wong M.W., Srinivasan M. (2023). Hybrid electrolyte design for high-performance zinc-sulfur battery. Small.

[12] Zhang L., Zhang M., Guo H., Tian Z., Ge L., He G., Huang J., Wang J., Liu T., Parkin I.P., Lai F. (2022). A universal polyiodide regulation using quaternization engineering toward high value-added and ultra-stable zinc-iodine batteries. Adv. Sci..

[13] Cao Y., Ju S., Zhang Q., Gao K., Marcelli A., Zhang Z. (2025). Recent progress in aqueous zinc-ion batteries based on conversion-type cathodes. Adv. Powder Mater..

[14] Gao L., Li Z., Zou Y., Yin S., Peng P., Shao Y., Liang X. (2020). A high-performance aqueous zinc-bromine static battery. iScience.

[15] Shahali H., Sellers R., Rafieerad A., Polycarpou A.A., Amiri A. (2024). Progress and prospects of zinc-sulfur batteries. Energy Storage Mater..

[16] Feng C., Jiang X., Zhou Q., Li T., Zhao Y., Niu Z., Wu Y., Zhou H., Wang M., Zhang X. (2023). Recent advances in aqueous zinc–sulfur batteries: overcoming challenges for sustainable energy storage. J. Mater. Chem. A.

[17] Bai Z., Wang G., Liu H., Lou Y., Wang N., Liu H., Dou S. (2024). Advancements in aqueous zinc-iodine batteries: a review. Chem. Sci..

[18] Chen H., Li X., Fang K., Wang H., Ning J., Hu Y. (2023). Aqueous zinc-iodine batteries: from electrochemistry to energy storage mechanism. Adv. Energy Mater..

[19] Liu J., Zhou W., Zhao R., Yang Z., Li W., Chao D., Qiao S.-Z., Zhao D. (2021). Sulfur-based aqueous batteries: electrochemistry and strategies. J. Am. Chem. Soc..

[20] Li W., Wang K., Jiang K. (2020). A low cost aqueous Zn-S battery realizing ultrahigh energy density. Adv. Sci..

[21] Wei C., Song J., Wang Y., Tang X., Liu X. (2023). Recent development of aqueous multivalent-ion batteries based on conversion chemistry. Adv. Funct. Mater..

[22] Liu J., Ye C., Wu H., Jaroniec M., Qiao S.-Z. (2023). 2D mesoporous zincophilic sieve for high-rate sulfur-based aqueous zinc batteries. J. Am. Chem. Soc..

[23] Zhang K., Wu C., Wang L., Ma C., Ye J., Wu Y. (2024). Polyethylene glycol-based colloidal electrode via water competition for ultra-stable aqueous Zn-I batteries. iScience.

[24] Wang Y.-H., Li X.-T., Wang W.-P., Yan H.-J., Xin S., Guo Y.-G. (2020). Chalcogen cathode and its conversion electrochemistry in rechargeable Li/Na batteries. Sci. China Chem..

[25] Zhang Z., Dong S., Cui Z., Du A., Li G., Cui G. (2018). Rechargeable magnesium batteries using conversion-type cathodes: A perspective and minireview. Small Methods.

[26] Kim J., Kim H., Kang K. (2018). Conversion-based cathode materials for rechargeable sodium batteries. Adv. Energy Mater..

[27] Xin S., Chang Z., Zhang X., Guo Y.-G. (2017). Progress of rechargeable lithium metal batteries based on conversion reactions. Natl. Sci. Rev..

[28] Wu F., Yushin G. (2017). Conversion cathodes for rechargeable lithium and lithium-ion batteries. Energy Environ. Sci..

[29] Kraytsberg A., Ein-Eli Y. (2017). A critical review-promises and barriers of conversion electrodes for Li-ion batteries. J. Solid State Electr..

[30] Guo X., Zhang S., Hong H., Wang S., Zhu J., Zhi C. (2025). Interface regulation and electrolyte design strategies for zinc anodes in high-performance zinc metal batteries. iScience.

[31] Jiang X., Wang Y., Li W., Lam Y., Zhang J., Bao X., Tang J., Zheng X., Jiang S.x., Jia H. (2025). High-performance Zn-I_2_ batteries enabled by porous hetero-carbon nanofiber hosts with TiO_2_ homojunctions. Adv. Fiber Mater..

[32] Du W., Song Z., Zheng X., Lv Y., Miao L., Gan L., Liu M. (2024). Recent progress on rechargeable Zn-X (X = S, Se, Te, I_2_, Br_2_) batteries. ChemSusChem.

[33] Yu X., Zhang T., Feng Y., Li X., Zhang J., Mi J., Ye C., Li W., Zhao D., Chao D. (2025). High-energy aqueous sulfur battery chemistry. Adv. Mater..

[34] Li P., Li C., Guo X., Li X., Zhi C. (2021). Metal-iodine and metal-bromine batteries: a review. Bull. Chem. Soc. Jpn..

[35] Chen Y., Ning J., Wen Y., Yao K., Zhang Y. (2025). Optimization strategies for high-performance aqueous zinc-sulfur batteries: challenges and future perspectives. Energy Mater..

[36] Wang S., Wu W., Jiang Q., Li C., Shi H.-Y., Liu X.-X., Sun X. (2025). Uncovering ZnS growth behavior and morphology control for high-performance aqueous Zn-S batteries. Chem. Sci..

[37] Zhao L., Yin D., Zhang Y., Li B., Wang S., Cui X., Feng J., Gao N., Liu X., Ding S., Zhao H. (2025). Hydrophobic ionic liquid enabled polyiodide confined transport in cathode realizing high areal capacity stable zinc-iodine battery. Energy Environ. Sci..

[38] Yang J., Song Y., Liu Q., Tang A. (2021). High-capacity zinc-iodine flow batteries enabled by a polymer-polyiodide complex cathode. J. Mater. Chem. A.

[39] Shao J., Li X., Zhang L., Qu Q., Zheng H. (2013). Core-shell sulfur@ polypyrrole composites as high-capacity materials for aqueous rechargeable batteries. Nanoscale.

[40] Li C., Yan L., Lv M., Wang M., Kong J., Bao W., Chang L. (2024). Spongy porous carbon nanosheets obtained by one-step oxidation-activation of asphalt with KHC_2_O_4_ activator: Application in the cathode of zinc storage devices. Sci. Total Environ..

[41] Lu K., Zhang H., Song B., Pan W., Ma H., Zhang J. (2019). Sulfur and nitrogen enriched graphene foam scaffolds for aqueous rechargeable zinc-iodine battery. Electrochim. Acta.

[42] Li W., Wang K., Jiang K. (2020). A low cost aqueous Zn-S battery realizing ultrahigh energy density. Adv. Sci..

[43] Cui M., Zhao H., Yin D., Gao N., Zhang Y., Zhao L., Wei Y., Liu M., Xi K., Ding S. (2024). Catalytical cobalt phthalocyanine/carbon nanotube cathode for high-performance zinc-iodine batteries. Energy Storage Mater..

[44] Sonigara K.K., Vaghasiya J.V., Mayorga-Martinez C.C., Pumera M. (2023). Flexible aqueous Zn-S battery based on an S-decorated Ti_3_C_2_T_x_ cathode. npj 2D Mater. Appl..

[45] Liu J., Chen S., Shang W., Ma J., Zhang J. (2025). In situ formation of 3D ZIF-8/MXene composite coating for high-performance zinc-iodine batteries. Adv. Funct. Mater..

[46] Guo X., Xu H., Tang Y., Yang Z., Dou F., Li W., Li Q., Pang H. (2024). Confining iodine into metal-organic framework derived metal-nitrogen-carbon for long-life aqueous zinc-iodine batteries. Adv. Mater..

[47] Chai L., Wang X., Hu Y., Li X., Huang S., Pan J., Qian J., Sun X. (2022). In-MOF-derived hierarchically hollow carbon nanostraws for advanced zinc-iodine batteries. Adv. Sci..

[48] Zhang P.F., Li J.H., Zhang S.J., Li D.C., Zeng S.Y., Xu S.L., Yao Q.X., Liu L.Y., Ding L., Li H.X. (2024). Toward shuttle-free Zn-I_2_ battery: anchoring and catalyzing iodine conversion by high-density P-doping sites in carbon host. Adv. Funct. Mater..

[49] Zhao S., Wu X., Zhang J., Li C., Cui Z., Hu W., Ma R., Li C. (2024). Biomass-derived porous carbon with single-atomic cobalt toward high-performance aqueous zinc-sulfur batteries at room temperature. J. Energy Chem..

[50] Ji Y., Xu J., Wang Z., Ren M., Wu Y., Liu W., Yao J., Zhang C., Zhao H. (2023). Nitrogen-doped litchi-shell derived porous carbon as an efficient iodine host for zinc-iodine batteries. J. Electroanal. Chem..

[51] He B., Li W.-C., Chen Z.-Y., Shi L., Zhang Y., Xia J.-L., Lu A.-H. (2021). Multilevel structured carbon film as cathode host for Li-S batteries with superhigh-areal-capacity. Nano Res..

[52] Yu D., Kumar A., Nguyen T.A., Nazir M.T., Yasin G. (2020). High-voltage and ultrastable aqueous zinc-iodine battery enabled by N-doped carbon materials: revealing the contributions of nitrogen configurations. ACS Sustainable Chem. Eng..

[53] Zeng S., Chen S., Ao Z., Lin X., Yan L., Liu C., Lin Z. (2025). Dual-mechanism anchoring of iodine species by pitch-derived porous carbon for enhanced zinc-iodine battery performance. Small.

[54] Xu C., Wang K., Zhang W., Fu F., Wang J. (2024). Evaluating the role of nitrogen in carbon hosts for aqueous zinc sulfur batteries. Ionics.

[55] Chen M., Zhu W., Guo H., Tian Z., Zhang L., Wang J., Liu T., Lai F., Huang J. (2023). Tightly confined iodine in surface-oxidized carbon matrix toward dual-mechanism zinc-iodine batteries. Energy Storage Mater..

[56] Hu L., Chen Y., Chen Y., Liu L., Liang S., Zhou N., Ding T., Jiang L., Wang L., Liang X., Hu K. (2024). Investigating the impact of mesoporous and microporous carbon host materials on the performance of sulfur cathodes in zinc-sulfur batteries. Mater. Lett..

[57] Yan L., Liu T., Zeng X., Sun L., Meng X., Ling M., Fan M., Ma T. (2022). Multifunctional porous carbon strategy assisting high-performance aqueous zinc-iodine battery. Carbon.

[58] Hou Y., Kong F., Wang Z., Ren M., Qiao C., Liu W., Yao J., Zhang C., Zhao H. (2023). High performance rechargeable aqueous zinc-iodine batteries via a double iodine species fixation strategy with mesoporous carbon and modified separator. J. Colloid Interface Sci..

[59] Li J., Liu J., Xie F., Bi R., Zhang L. (2024). Synergistic electrocatalysis and spatial nanoconfinement to accelerate sulfur conversion kinetics in aqueous Zn-S battery. Angew. Chem..

[60] Feng W., Wang Y., Tian F., Liu Z., Wei X., Ma C., Ma G., Li Z., Kong D., Zhi L. (2024). Enhanced carbon host with N-reinforced S-sites to catalyze rapid iodine conversion kinetics for Zn-I_2_ battery. Energy Storage Mater..

[61] Dai P., Lang J., Huang W., Ma L., Zhao X., Lin X., Li Q., Li H., Liu T., Amine K., Li H. (2025). Spin state modulation via magnetic fields in Fe single atom catalysts for high-performance aqueous zinc-sulfur batteries. ACS Nano.

[62] Hei P., Sai Y., Li W., Meng J., Lin Y., Sun X., Wang J., Song Y., Liu X.X. (2024). Diatomic catalysts for aqueous zinc-iodine batteries: mechanistic insights and design strategies. Angew. Chem. Int. Ed. Engl..

[63] Gao Y., Chen C., Zhang J., Chen M., Shan L., Luo Q., Xing Z., Zhao Z., Li J., Rao P. (2025). Integrated confinement-chemisorption-catalysis cathode for highly stable zinc-iodine batteries. Nano Mater. Sci..

[64] Li S., Sun C., Zhang M., Tang R., Chen M., Meng W., Yang J., Kang Y., Lv Z., Zhao J., Yang Y. (2025). An integrated tandem-structured separator enables dual-enhanced stable interfaces for long-cycle-life and high-areal-capacity aqueous zinc-iodine batteries. Angew. Chem. Int. Ed. Engl..

[65] Yang J., Kang Y., Meng F., Meng W., Chen G., Zhang M., Lv Z., Wen Z., Li C.C., Zhao J., Yang Y. (2025). Theoretical calculation-driven rational screening of d-block single-atom electrocatalysts based on d-p orbital hybridization for durable aqueous zinc–iodine batteries. Energy Environ. Sci..

[66] Yang F., Long J., Yuwono J.A., Fei H., Fan Y., Li P., Zou J., Hao J., Liu S., Liang G. (2023). Single atom catalysts for triiodide adsorption and fast conversion to boost the performance of aqueous zinc-iodine batteries. Energy Environ. Sci..

[67] Zhang H., Shang Z., Luo G., Jiao S., Cao R., Chen Q., Lu K. (2022). Redox catalysis promoted activation of sulfur redox chemistry for energy-dense flexible solid-state Zn-S battery. ACS Nano.

[68] Chen Q., Chen S., Ma J., Ding S., Zhang J. (2023). Synergic anchoring of Fe_2_N nanoclusters on porous carbon to enhance reversible conversion of iodine for high-temperature zinc-iodine battery. Nano Energy.

[69] Jiang X., Zhou Y., Wang Y., Teng J., Wang K., Zhang J., Li W., Liu G., Fu S., Jia H. (2025). Stabilizing zinc-iodine batteries via amyloid fibril-based electrolytes: ion transport and pH regulation through hierarchical networks. Adv. Funct. Mater..

[70] Xu Z., Zhang Y., Gou W., Liu M., Sun Y., Han X., Sun W., Li C. (2022). The key role of concentrated Zn(OTF)_2_ electrolyte in the performance of aqueous Zn-S batteries. Chem. Commun..

[71] Zhao Y., Wang D., Li X., Yang Q., Guo Y., Mo F., Li Q., Peng C., Li H., Zhi C. (2020). Initiating a reversible aqueous Zn/sulfur battery through a “liquid film”. Adv. Mater..

[72] Luo L.-W., Zhang C., Wu X., Han C., Xu Y., Ji X., Jiang J.-X. (2021). A Zn-S aqueous primary battery with high energy and flat discharge plateau. Chem. Commun..

[73] Zhang K., Yu Q., Sun J., Tie Z., Jin Z. (2024). Precipitated iodine cathode enabled by trifluoromethanesulfonate oxidation for cathode/electrolyte mutualistic aqueous Zn-I batteries. Adv. Mater..

[74] Qian M., Lei J., Hao M., Li Q., Zhan J., Tang F., Wu F. (2025). Choline iodide-mediated sulfur conversion and zinc plating/stripping chemistry in aqueous Zn-S batteries. Angew. Chem..

[75] Liu X., Tao R., Huang G., Yang Y., Wang H., Yuan H., Wang D., Liu Z., Liu J., Liang J. (2025). Deciphering failure mechanisms of Zn-S batteries: anion-cation synergy for dual-interface stabilization toward dendrite-free zinc and reversible sulfur conversion. Energy Environ. Sci..

[76] Chen Z., Gao X., Shan L., Fu Q., Xing Z., Rao P., Kang Z., Shi X., Zhang W., Tian X. (2025). Taming polyiodides: phenol chemistry for shuttle-free and durable zinc-iodine batteries. Energy Environ. Sci..

[77] Chang G., Liu J., Hao Y., Huang C., Yang Y., Qian Y., Chen X., Tang Q., Hu A. (2023). Bifunctional electrolyte additive with redox mediation and capacity contribution for sulfur cathode in aqueous Zn-S batteries. Chem. Eng. J..

[78] Li W., Wang K., Jiang K. (2020). A low cost aqueous Zn-S battery realizing ultrahigh energy density. Adv. Sci..

[79] Wu W., Wang S., Lin L., Shi H.-Y., Sun X. (2023). A dual-mediator for a sulfur cathode approaching theoretical capacity with low overpotential in aqueous Zn-S batteries. Energy Environ. Sci..

[80] Wang M., Meng Y., Sajid M., Xie Z., Tong P., Ma Z., Zhang K., Shen D., Luo R., Song L. (2024). Bidentate coordination structure facilitates high-voltage and high-utilization aqueous Zn-I_2_ batteries. Angew. Chem..

[81] Wang F., Liang W., Liu X., Yin T., Chen Z., Yan Z., Li F., Liu W., Lu J., Yang C., Yang Q. (2024). A bifunctional electrolyte additive features preferential coordination with iodine toward ultralong-life zinc-iodine batteries. Adv. Energy Mater..

[82] Hei P., Sai Y., Yu L., Lin Y., Li B., Hu G., Wu W., Wang J., Sun X., Liu X.-X., Song Y. (2025). Cosolvent electrolyte design for high-voltage aqueous zinc-sulfur batteries. J. Am. Chem. Soc..

[83] Guo Y., Zhu X., Zhang J., Zhang T., Wang Z., Shan M., Wang F., Cao C.C., Xu G., Zhu M. (2025). Engineering electrolyte network structure for improved kinetics and dendrite suppression in Zn-S batteries. Angew. Chem. Int. Ed. Engl..

[84] Yang M., Yan Z., Xiao J., Xin W., Zhang L., Peng H., Geng Y., Li J., Wang Y., Liu L., Zhu Z. (2022). Boosting cathode activity and anode stability of Zn-S batteries in aqueous media through cosolvent-catalyst synergy. Angew. Chem. Int. Ed. Engl..

[85] Yan Y., Jiao Y., Wu P. (2025). Realizing high performance four-electron zinc-iodine batteries with acidic eutectic electrolyte. Angew. Chem..

[86] Yang J.L., Xiao T., Xiao T., Li J., Yu Z., Liu K., Yang P., Fan H.J. (2024). Cation-conduction dominated hydrogels for durable zinc-iodine batteries. Adv. Mater..

[87] Ji Y., Xie J., Shen Z., Liu Y., Wen Z., Luo L., Hong G. (2023). Advanced zinc-iodine batteries with ultrahigh capacity and superior rate performance based on reduced graphene oxide and water-in-salt electrolyte. Adv. Funct. Mater..

[88] Yang H., Qiao Y., Chang Z., Deng H., He P., Zhou H. (2020). A metal-organic framework as a multifunctional ionic sieve membrane for long-life aqueous zinc-iodide batteries. Adv. Mater..

[89] Xu M., Wei L., Zhao G., Wang Y., Yu D. (2025). Synergistic adsorption-catalysis strategy enables multifunctional separator for high-performance Zn-I_2_ batteries. Colloid. Surface. A..

[90] Zhang Y., Zeng Z., Yang S., Zhang Y., Ma Y., Wang Z. (2023). A nanocellulose-mediated, multiscale ion-sieving separator with selective Zn^2+^ channels for durable aqueous zinc-based batteries. Energy Storage Mater..

[91] Zhang Y., Yao M., Jing P., Fu Y., Wang W., Zhang Y., Lai Q., Wang Q. (2025). Achieving a highly reversible four-electron redox of S/Cu_2_S for aqueous Zn/S-Cu battery. Angew. Chem. Int. Ed. Engl..

[92] Qiu C., Chen M., Pan Y., Shi X., Yang Y., Li F., Xing Z., Li J., Zhao Z., Shan L., Tian X. (2025). Unlocking high-performance four-electron zinc-iodine batteries through halogen bonding inversion and non-identical-frequency molecular vibrations. Angew. Chem..

[93] Chen M., Chen G., Sun C., Li X., Zhang M., Hua H., Zhao J., Yang Y. (2025). SiO_2_ nanoparticles-induced antifreezing hydrogel electrolyte enables Zn-I_2_ batteries with complete and reversible four-electron-transfer mechanisms at low temperatures. Angew. Chem..

[94] Zou Y., Liu T., Du Q., Li Y., Yi H., Zhou X., Li Z., Gao L., Zhang L., Liang X. (2021). A four-electron Zn-I_2_ aqueous battery enabled by reversible I^−^/I_2_/I^+^ conversion. Nat. Commun..

[95] Ma W., Liu T., Xu C., Lei C., Jiang P., He X., Liang X. (2023). A twelve-electron conversion iodine cathode enabled by interhalogen chemistry in aqueous solution. Nat. Commun..

[96] Ran L., Cai X., Ma D., Zhao J., Zhang H., Liang S., Tao J., Zhang X., Li Y., Fan H.J., Song W. (2025). Stable four-electron zinc-iodine battery realized by polyacrylamide as catalytic binder. Angew. Chem. Int. Ed. Engl..

[97] Liu T., Lei C., Wang H., Li J., Jiang P., He X., Liang X. (2024). Aqueous electrolyte with weak hydrogen bonds for four-electron zinc-iodine battery operates in a wide temperature range. Adv. Mater..

[98] Liu T., Lei C., Wang H., Xu C., Ma W., He X., Liang X. (2024). Practical four-electron zinc-iodine aqueous batteries enabled by orbital hybridization induced adsorption-catalysis. Sci. Bull..

[99] Wang Y., Lv Y., Wei S., Yu L., Yuan B., Shifa T.A., Muhammad M., Wang R., Li J., Zhao Y., Sun X. (2025). Polymerized melamine catalyzes direct I^−^/I_2_ conversion via ─N═N─ motif for high capacity Zn-I_2_ batteries. Adv. Mater..

[100] Yan Y., Jiao Y., Wu P. (2025). Realizing high performance four-electron zinc-iodine batteries with acidic eutectic electrolyte. Angew. Chem..

[101] Du W., Huang Q., Zheng X., Lv Y., Miao L., Song Z., Gan L., Liu M. (2025). High-conversion-efficiency and stable six-electron Zn-I_2_ batteries enabled by organic iodide/thiazole-linked covalent organic frameworks. Energy Environ. Sci..

